# Innate Immune-Modulatory Activity of *Prunella vulgaris* in Thyrocytes Functions as a Potential Mechanism for Treating Hashimoto’s Thyroiditis

**DOI:** 10.3389/fendo.2020.579648

**Published:** 2020-11-16

**Authors:** Fei Chen, Akira Kawashima, Yuqian Luo, Mitsuo Kiriya, Koichi Suzuki

**Affiliations:** ^1^ Department of Clinical Laboratory Science, Faculty of Medical Technology, Teikyo University, Tokyo, Japan; ^2^ Department of Thyroid Surgery, Zhujiang Hospital, Southern Medical University, Guangzhou, China; ^3^ Department of Laboratory Medicine, Nanjing Drum Tower Hospital and Jiangsu Key Laboratory for Molecular Medicine, Nanjing University Medical School, Nanjing, China

**Keywords:** *Prunella vulgaris*, innate immune response, autoimmune thyroid diseases, dsDNA, dsRNA

## Abstract

*Prunella vulgaris* (PV), a perennial herb, has been used to treat thyroid diseases in China for over 2,000 years. In particular, its therapeutic effect has been described for Hashimoto’s thyroiditis, including reducing titers autoantibodies against thyroid peroxidase and thyroglobulin of and T helper 17 (Th17) cells. However, the underlying mechanism for how PV exerts such effects has not been investigated. We examined the effects of PV on innate immune activation, which is thought to be one of the triggers for the development of autoimmune diseases, including Hashimoto’s thyroiditis. In cultured thyrocytes, PV reduced mRNA levels of inflammatory cytokines that were originally induced as a result of innate immune activation initiated by transfection of double-stranded DNA (dsDNA) or dsRNA. PV suppressed activation of nuclear factor κB (NF-κB) and interferon regulatory factor 3 (IRF3), and suppressed corresponding promoter activation, which were initially activated by dsDNA or dsRNA. PV also suppressed the mRNA levels of molecules responsible for antigen processing and presentation, and PV protected thyrocytes from apoptosis induced by dsDNA and dsRNA. Additionally, PV suppressed the expression of genes involved in iodide uptake and oxidation. Taken together, these results suggest that PV exerts its protective effect on thyrocytes by suppressing both innate and adaptive immune responses and cell death. PV may also protect cells from iodide-associated oxidative injury. This report is among the first to identify the mechanisms to explain PV’s beneficial effects in Hashimoto’s thyroiditis.

## Introduction

*Prunella vulgaris* (PV) is an herbaceous plant in the genus *Prunella*. The young leaves and stems can be eaten raw in salads, and the spikes are dried, powdered and brewed for use in beverages or as herbal medicine. PV’s anti-inflammatory and immunomodulatory effects have been recognized during the long-term practice of traditional Chinese medicine (1). It is prescribed to treat headache, vertigo, mastitis, hyperplasia of mammary glands, lymphadenopathy, hyperthyroidism and thyroid goiters, in forms of topical ointments, oral liquid and capsules (2, 3). Additionally, PV in liquid or in capsules, in combination with Western medicines (e.g. levothyroxine, indomethacin or prednisone), has been used to treat patients with Hashimoto’s thyroiditis. It has been shown that PV significantly improved titers of TPO-Ab and TG-Ab, and the proportion of T helper 17 (Th17) cells among CD4^+^ T cell populations, compared with Western medicines alone (4–8). However, the underlying mechanisms for its therapeutic effects are poorly understood.

Hashimoto’s thyroiditis is an autoimmune disease characterized by chronic thyroiditis with severe parenchymal infiltration of lymphocytes. Although the precise pathologic mechanisms are not fully understood, it is generally believed that the disease manifests through a combination of genetic susceptibility and environmental risk factors (9). Recent studies suggest that innate immune responses in thyrocytes triggered by pathogen-associated molecular patterns (PAMPs; characteristic of harmful foreign bacteria, virus or fungi) and/or danger-associated molecular patterns (DAMPs; typically, ectopic exposure of tissue injury-derived self-nucleotides, proteins or free oxygen radicals) are initiating events for Hashimoto’s thyroiditis (10, 11).

Recognition of PAMPs or DAMPs by thyrocytes causes the activation of innate immune responses, which is characterized by the production of an array of inflammatory mediators, such as tumor necrosis factor α (TNF-α), interleukin-6 (IL-6) and interferon-β (IFN-β) (10, 11). These immune mediators recruit lymphocytes to the inflamed site, which putatively increases the likelihood of breaking self-tolerance, particularly in genetically susceptible individuals. Moreover, the inflamed thyrocytes are functionally suppressed or even undergo cell death, which may directly precipitate thyroid destruction and hypothyroidism (12, 13). Therefore, agents that interfere with innate immune activation in thyrocytes may be able to improve thyroiditis.

In the current study, we investigated potential immunomodulatory effects of PV on innate immune response in PAMP/DAMP-stimulated rat thyroid FRTL-5 cells. We used double-stranded DNA (dsDNA) as a model of both PAMPs (DNA viruses and bacteria) (14–16) and DAMPs (self-DNA fragments from injured cells) (10, 12, 14), and dsRNA as a model of PAMPs (RNA viruses). We also studied the effect of PV on the thyroid-specific functional gene expressions in FRTL-5 cells.

## Materials and Methods

### Preparation of Aqueous Extraction of PV

Aqueous extraction of PV was prepared and used as previously reported (17–19). In brief, a fine powder of 40 g of PV was mixed with 400 mL of H_2_O and boiled for 2 h in a glass beaker. The boiled extracts were centrifuged at 20,000 rpm for 10 min to remove debris and the supernatant was further powdered in a rotary vacuum evaporator under 10 mbar at 70°C for 5 h. The powdered PV extract was weighed and dissolved in H_2_O to a stock concentration of 50 mg/mL, then filtrated through 0.22 μm PES membrane (Merck Millipore, Darmstadt, Germany) and stored at −80°C for future use.

### Cell Culture and Treatment

FRTL-5 rat thyroid cells were grown in Coon’s modified Ham’s F-12 medium containing 5% heat-treated bovine serum (Invitrogen, Carlsbad, CA) and a mixture of six hormones, including bovine TSH (1 mU/mL), insulin (10 μg/mL), hydrocortisone (0.36 ng/mL), transferrin (5 μg/mL), Gly-His-Lys-acetate (2 ng/mL), and somatostatin (10 ng/mL) as described (20). All reagents were purchased from Sigma-Aldrich (St. Louis, MO). PV was used at concentrations of 0, 31.25, 62.5, 125, 250, 500 μg/mL in culture medium.

### Transfection of Nucleic Acids

One microgram of synthetic polynucleotides, i.e. poly (dA:dT) and poly(I:C) (GE Healthcare, Little Chalfont, UK), was mixed with 3 μL of Fugene HD transfection reagent (Roche Diagnostics, Basel, Switzerland) and 100 μL of serum-free cell culture medium, and then incubated for 15 min at room temperature. The solution was added to the cells and incubated for 6 h at 37°C in a CO_2_ incubator after which the medium was replaced with regular culture medium containing 5% bovine serum.

### RNA Purification and Real-Time PCR

Total RNA was isolated using RNeasy Plus Mini Kit (Qiagen, Hilden, Germany), and cDNA was synthesized using the High-Capacity cDNA Reverse Transcription Kits (Applied Biosystems, Waltham, MA) as described previously (20). Real-time PCR was performed using Fast SYBR Green Master Mix (Applied Biosystems) according to the manufacturer’s instructions. A total of 20 ng of cDNA mixed with 20 μL of FastStart Universal SYBR Green Master (Roche Diagnostics) was amplified by incubating for 30 s at 95°C, followed by 40 cycles of 5 s at 95°C and 30 s at 60°C, and one cycle of 15 s at 95°C, 30 s at 60°C, and 15 s at 95°C. The mRNA levels were normalized against that of *Gapdh* levels using the ΔΔCt method as described (20). The primers used were as follows: *Gapdh* forward, 5′-ACAGCAACAGGGTGGTGGAC-3′; *Gapdh* reverse, 5′-TTTGAGGGTGCAGCGAACTT-3′; *Tpo* forward, 5′-CACGGCTTACCAGGCTACAA-3′; *Tpo* reverse, 5′-GCCTCCCAACCAGACATCAA-3′; *Duox2* forward, 5′-CAGCGCTACGACGGCTGGTTTA-3′; *Duox2* reverse, 5′-CCCAAGCACTGTGCGGTTGT-3′; *Duoxa2* forward, 5′-TCAGCGTACCGCTGCTCATCGT-3′; *Duoxa2* reverse, 5′-ACCAACCAGAACCAGCGCGAGT-3′; *Slc5a5* forward, 5′-CTACCGTGGGTGGTATGAAGG-3′; *Slc5a5* reverse, 5′-TGCCACCCACTATGAAAGTCC-3′; *Tnfa* forward, 5′-ATGGGCTCCCTCTCATCAGT-3′; *Tnfa* reverse, 5′-GCTTGGTGGTTTGCTACGAC-3′; *Il6* forward, 5′-AGCGATGATGCACTGTCAGA-3′; *Il6* reverse, 5′-GGAACTCCAGAAGACCAGAGC-3′; *Ifnb* forward, 5′-CTTGGGTGACATCCACGACT-3′; *Ifnb* reverse, 5′-AAGACTTCTGCTCGGACCAC-3′.

### Transient Transfection of Plasmids and Reporter Gene Assays

FRTL-5 cells (1x10^5^) plated on poly-D-lysine coated 24-well plates (Greiner Bio One, FL) were transfected with 0.2 μg of either NF-κB-dependent luciferase reporter plasmid (p5NF-κB-luc) or rat IFN-β-dependent promoter luciferase reporter plasmid (pGL3-IFNβ) using Fugene HD transfection reagent (Roche Diagnostics) in serum-free medium. The pGL3-basic plasmid was used as a control. After transfection for 6 h, the medium was replaced with basal medium, and cells were treated with PV extract for 24 h. Cells were then stimulated with either dsDNA or dsRNA for 6 h, and a reporter gene assay was performed using the Bright-Glo Luciferase assay system (Promega, Madison,WI). Luciferase activities were measured using FLUO star galaxy (BMG Labtech, Offenburg, Germany), and were normalized to corresponding protein concentrations that were determined using Bio-Rad DC Protein Assay Kits (Bio-Rad Laboratories, Hercules, CA). Data was expressed relative to the activity of the control group.

### Protein Preparation and Western Blot Analysis

Cells were lysed in a buffer containing 150 mM NaCl, 1% Nonidet P-40, 0.5% sodium deoxycholate, 0.1% SDS and 50 mM Tris, pH 8.0 for 1 h. The supernatant was collected after centrifugation, and 6 μg of protein was used for Western blotting. Briefly, the proteins were separated on NuPage 4–12% Bis-Tris gels (Invitrogen) by electrophoresis and transferred to nitrocellulose i-Blot gel transfer stacks (Invitrogen). The membrane was washed with PBS with 0.1% Tween 20 (PBST), placed in blocking buffer (PBST containing 5% nonfat milk) for 1 h. Then the membrane was incubated with primary antibodies at 4°C overnight. The primary antibodies used were rabbit anti-nuclear factor of kappa light polypeptide gene enhancer in B-cells inhibitor α (IκB-α) (Cell Signaling Technology, Danvers, MA; 1:1,000), phosphorylation interferon regulatory factor 3 (pIRF3) (Cell Signaling Technology; 1:1,000), and mouse monoclonal anti-β-actin (Sigma; 1:5,000) as an internal control. After washing with PBST, membranes were incubated with a horseradish peroxidase (HRP)-labeled goat anti-mouse IgG (Cell Signaling Technology; 1:5,000) or goat anti-rabbit IgG (Cell Signaling Technology; 1:1,000) as secondary antibodies. Specific proteins were visualized using Immunostar LD reagent (Wako Pure Chemical, Osaka, Japan), and the chemiluminescence was detected using the C-DiGit blot scanner (LI-COR).

### Fluorescence Staining

FRTL-5 cells were cultured on glass-bottom dishes (Matsunami Glass, Osaka, Japan) in the presence or absence of 350 μg/mL of PV for 24 h. Cells were then stimulated with 1 μg/mL dsDNA or dsRNA for 24 h and stained with Hoechst 33342. Fluorescence was visualized and the images were captured on an FV10i confocal laser scanning microscope (Olympus, Tokyo, Japan).

### Statistical Analysis

All experiments were repeated at least three times using different batches of cells, and the mean ± SD was calculated. The significance of the differences between experimental values was determined by an unpaired two-tailed t-test, with *p* < 0.05 considered to be significant.

## Results

### PV Abolishes mRNA Expression of Inflammatory Cytokines Induced by dsDNA or dsRNA

Inflammatory cytokines including IL-6, TNF-α and IFN-β have been found extensively in both thyroids and sera of patients with Hashimoto’s thyroiditis (21, 22). These molecules recruit lymphocytes into the affected sites to precipitate inflammation while interfering with thyroid hormone synthesis (23), thus affecting the balance between the maintenance of self-tolerance and the initiation of autoimmunity. In addition to the infiltrating T cells, we previously showed that the thyrocytes themselves can produce various cytokines in response to dsDNA or dsRNA (10, 12, 14), thus playing an active role in thyroid inflammation and the development of autoimmunity.

**Figure 1:**
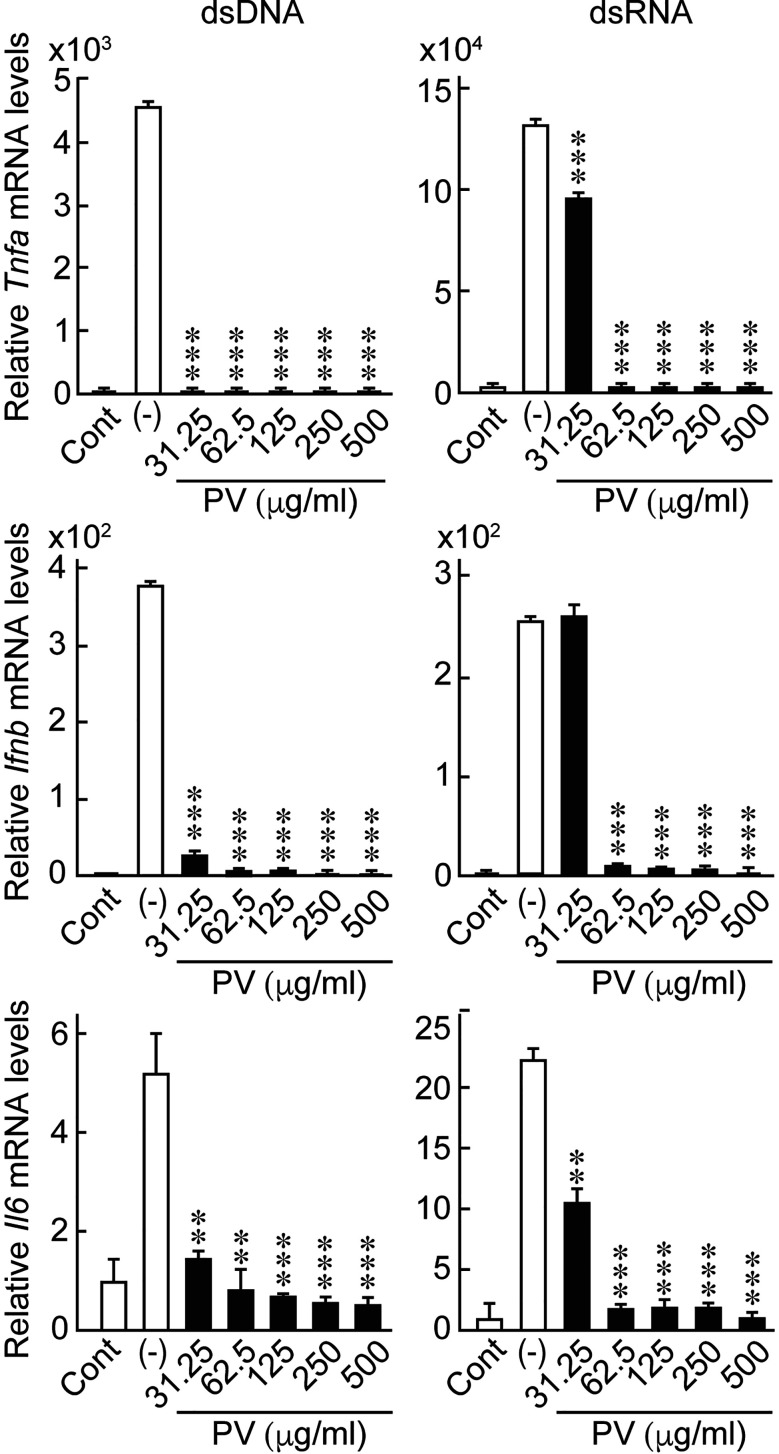
PV suppresses the mRNA expression of inflammatory cytokines induced by dsDNA or dsRNA. FRTL-5 cells were pretreated with PV at increasing concentrations (0, 31.25, 62.5, 125, 250, 500 μg/mL) for 24 h. Cells were then stimulated with 1 μg of dsDNA or dsRNA for 24 h in the presence of absence of PV. Total RNA was purified and subjected to real-time PCR analysis to determine the relative mRNA expression levels of *Tnfa, Ifnb and Il6.* mRNA levels were normalized against *Gapdh* levels and expressed as fold-change relative to the control. Data are presented as mean ± SD (n = 3) relative to the mRNA levels of non-treated control cells. **p < 0.01 and ***p < 0.001, compared to the PV (−) value.

In order to investigate the possible effects of PV on innate immune responses induced in thyrocytes, FRTL-5 cells were stimulated with dsDNA or dsRNA in the presence or absence of PV. The concentrations of PV used in this study is approximately equal to 15-230-fold dilution of its daily dosage for a patient. No cytotoxic effect of PV was noticed within this range, as determined by trypan blue exclusion test of cell viability (**Supplementary Figure 1**). Real-time PCR analysis showed that mRNA levels of *Ifnb*, *Tnfa* and *Il6* were significantly upregulated 24 h after stimulation with dsDNA or dsRNA (**Figure 1**). However, the increase in the expression of inflammatory cytokines was remarkably reversed by PV in a dose-dependent manner (**Figure 1**), indicating that PV exerts a powerful suppressive effect on innate immune activation in thyrocytes. mRNA levels of *Gapdh* were not affected by PV (data not shown).

### PV Suppresses the Activation of Nuclear Factor κB (NF-κB) and Interferon Regulatory Factor 3 (IRF3), With Suppression of Their Downstream Corresponding Promoters

**Figure 2:**
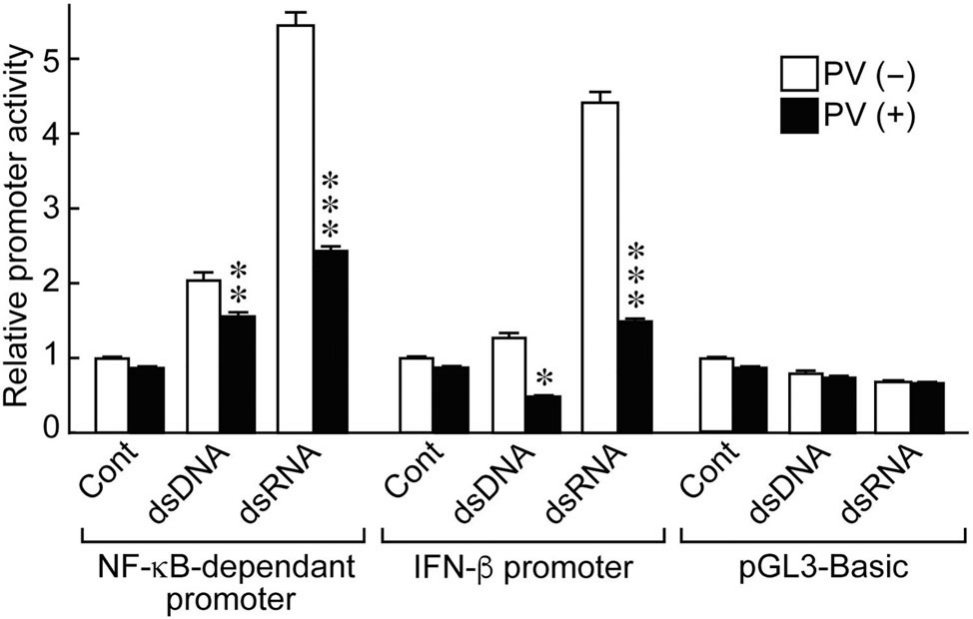
PV suppresses NF-κB-dependent and IFN-β-dependent promoter activation stimulated by dsDNA or dsRNA. FRTL-5 cells were transfected with luciferase chimeric plasmids containing an NF-κB-dependent promoter (p5NF-κB-luc), an IFN-β promoter (pGL3-IFN-β), or a basic vector (pGL3-basic) for 6 h. Cells were then treated with 350 μg/mL of PV for 24 h, and stimulated with 1 μg of dsDNA or dsRNA for another 24 h. Luciferase reporter gene assays were performed as described in the Materials and Methods. Data are the results from three different experiments, each performed in triplicate, and expressed as the mean ± SD. *p < 0.05, **p < 0.01 and ***p < 0.001, compared to PV (−) value.

NF-κB and IFN-β are essential inducers of series of inflammatory cytokines upon activation of innate immunity (24, 25). To determine if PV affects cytokine transcription, we performed luciferase reporter gene assays using NF-κB- and IFN-β-dependent promoter constructs. Stimulating the cells with dsRNA induced NF-κB-dependent promoter activation, as well as IFN-β promoter activation (**Figure 2**). However, PV significantly suppressed the promoter activation of NF-κB- and IFN-β-dependent promoter constructs in both dsDNA- and dsRNA-stimulated cells (**Figure 2**).

**Figure 3:**
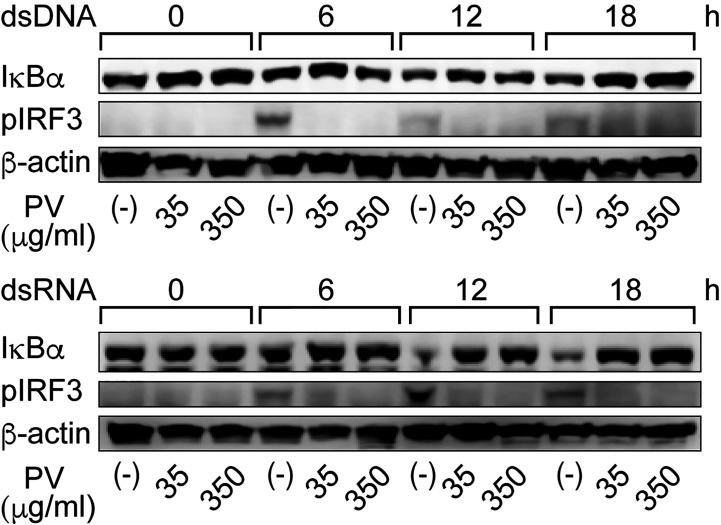
PV suppresses degradation of IκB-α and phosphorylation of IRF3 induced by dsDNA or dsRNA. FRTL-5 cells were pretreated with PV at 35 μg/mL or 350 μg/mL for 24 h, then stimulated with 1 μg of dsDNA or dsRNA for the indicated period of time. Whole cell proteins were extracted, and Western blot analysis was performed to determine the protein expression levels of IκB-α, pIRF3 and β-actin.

It is known in unstimulated cells that NF-κB is sequestered in an inactive state in the cytoplasm by binding with a family of inhibitors, termed inhibitor of κB (IκB), and activation of NF-κB is initiated by signal-induced degradation of IκB proteins (25). IRF3 is known as another major regulator that plays an important role in innate immune response (24). In unstimulated cells, IRF3 is found in an inactive cytoplasmic form, and upon serine/threonine phosphorylation, it translocates to the nucleus and activates the transcription of type I IFNs as well as other IFN-inducible genes (24). Therefore, we examined protein levels of IκB-α, the major subtype of IκB, and phosphorylated IRF3 (pIRF3) by Western blot analysis. When FRTL-5 cells were stimulated by dsDNA or dsRNA, the levels of IκB-α protein were decreased at 12 h and 18 h, whereas pIRF3 was rather induced in the absence of PV (**Figure 3**). However, PV reversed the effects of dsDNA and dsRNA to keep IκB-α and pIRF3 levels almost to the levels seen prior to stimulation with dsDNA or dsRNA (**Figure 3**). These results together suggest that PV interferes with NF-κB and IRF3 signaling pathways, which were initiated by dsDNA or dsRNA in thyrocytes, indicating at least two molecular pathways underlying the protective innate immune response-suppressive effect of PV.

### PV Inhibits Genes Related to Antigen Presentation Pathways Induced by dsDNA or dsRNA

**Figure 4:**
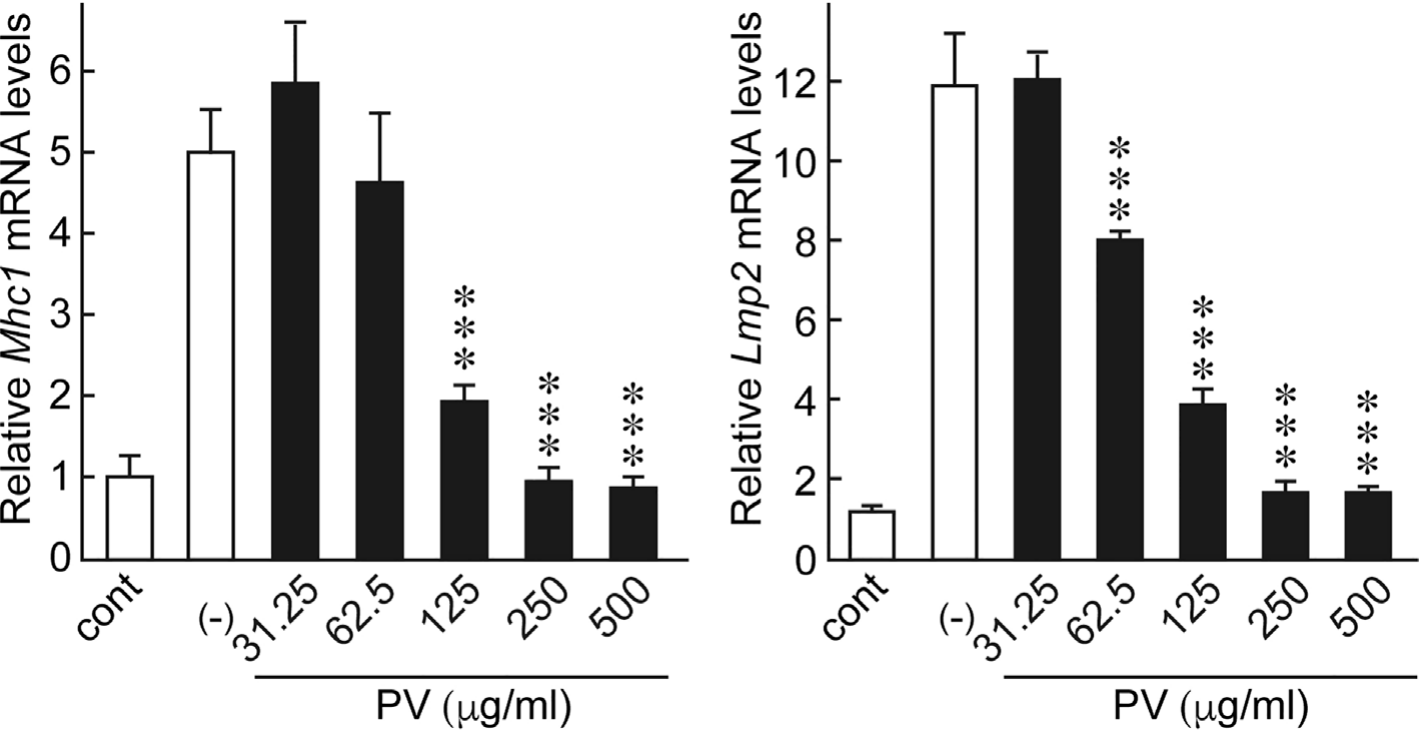
PV inhibited genes involved in antigen presentation pathways induced by dsRNA. FRTL-5 cells were pretreated with PV at increasing concentrations (0, 31.25, 62.5, 125, 250, 500 μg/mL) for 24 h. Cells were then stimulated with 1 μg of dsRNA for 24 h in the presence of PV. Total RNA was purified from the cells and subjected to real-time PCR analysis to determine the relative mRNA expression levels of *Mhc1* and *Lmp2.* mRNA levels were normalized against *Gapdh* levels and expressed as fold-change relative to the control. Data are presented as mean ± SD (n = 3) relative to the mRNA levels of non-treated control levels. ***p < 0.001, compared to PV (−) value.

Innate immune activation and inflammatory cytokine production theoretically activates the adaptive immune system, which increases the chance of breaking tolerance to immunogenic self-antigens, eventually leading to the development of thyroid autoimmunity. Moreover, the induction of genes related to antigen presentation pathways in thyrocytes is putatively another important mechanism by which the innate immune system mobilizes the adaptive immune system (10, 12, 14–16). In FRTL-5 cells, dsRNA stimulation significantly induced the gene expression of major histocompatibility complex class I (*Mhc1*) and low molecular weight protein 2 (*Lmp2*), which are involved in the MHC class I antigen processing pathway. Therefore, we evaluated the effect of PV on mRNA levels of *Mhc1* and *Lmp2* induced by dsRNA. We found that PV inhibited dsRNA-induced *Mhc1* and *Lmp2* mRNA levels in a dose-dependent manner in FRTL-5 cells (**Figure 4**), revealing another aspect of the versatile immunomodulatory effects of PV that may hinder autoantigen presentation.

### PV Exerts a Protective Effect on Inflammation-Associated Cell Death Induced by dsDNA or dsRNA in Thyrocytes

**Figure 5:**
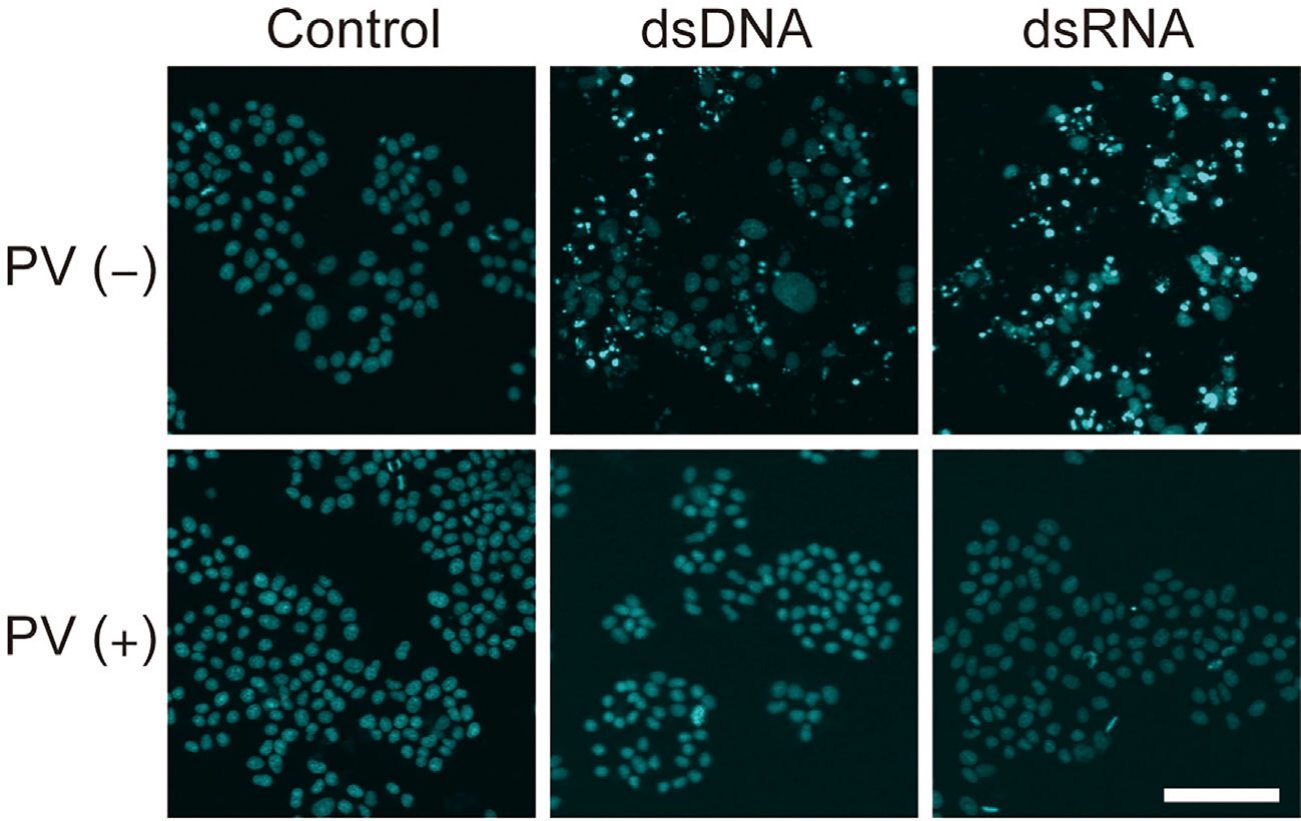
PV exerts a protective effect on cell death in FRTL-5 cells induced by dsDNA or dsRNA. FRTL-5 cells were cultured with or without PV at 350 μg/mL for 24 h. Cells were then stimulated with 1 μg/mL dsDNA or dsRNA for 24 h, followed by Hoechst 33342 staining. Fluorescence was observed using a confocal laser scanning microscope. Scale bar, 100 µm.

Inflammatory responses induce apoptosis, which is also seen in Hashimoto’s thyroiditis. After exposure to dsDNA or dsRNA, a majority of FRTL-5 thyroid cells exhibited condensed, fragmented nuclei with a much brighter color, indicative of cellular apoptosis, as revealed by Hoechst 33342 staining [**Figure 5**, **PV** (**−**)]. However, PV completely prevented such apoptotic changes [**Figure 5**, **PV** (**+**)], indicating that PV is a powerful protective agent against inflammation-related cell death in thyrocytes.

### PV Suppresses Gene Expression Involved in Iodide Uptake and Oxidation

**Figure 6:**
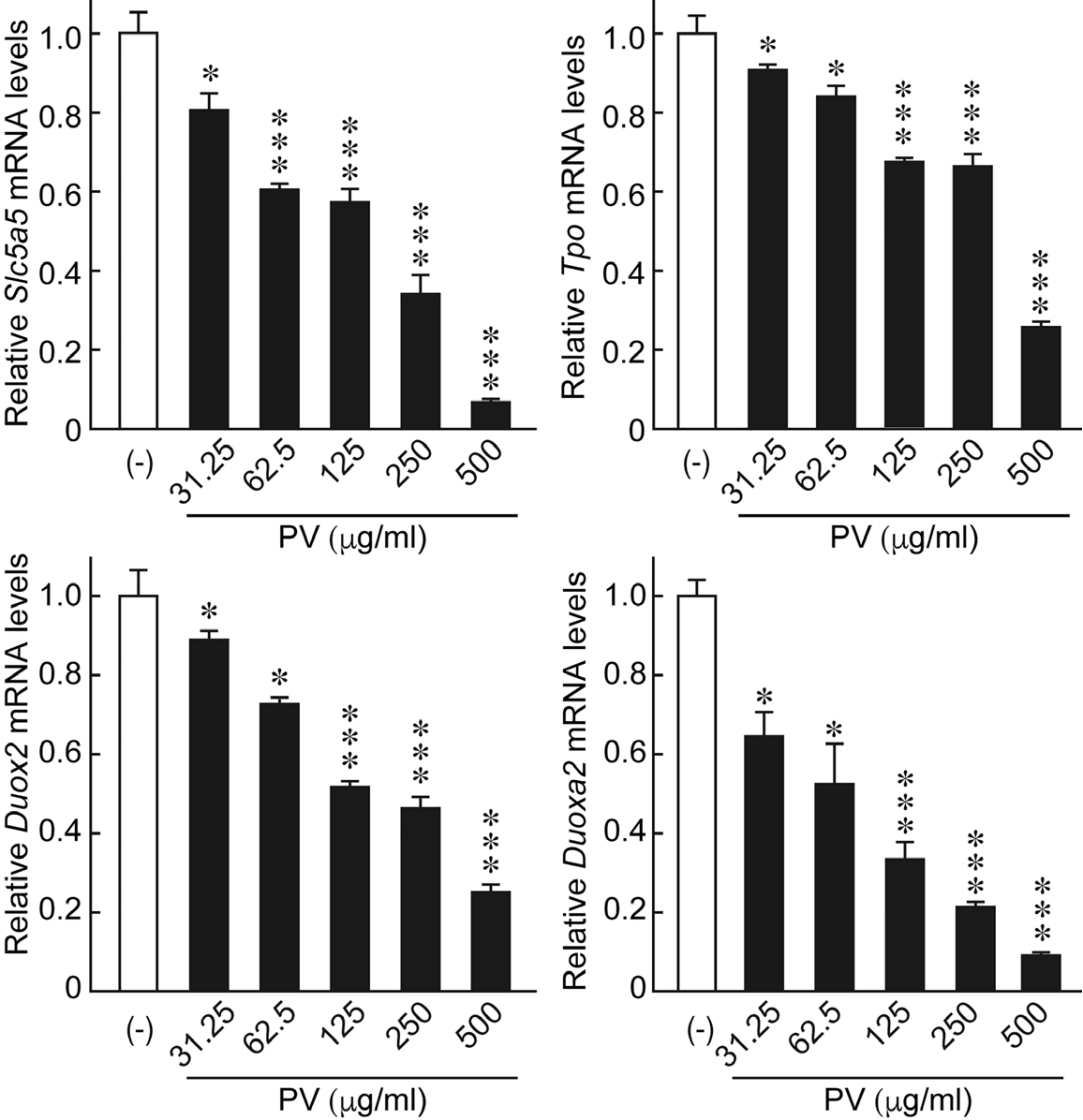
PV suppresses the mRNA expression of genes essential for thyroid hormone biosynthesis. FRTL-5 cells were treated with PV at increasing concentrations (0, 31.25, 62.5, 125, 250, 500 μg/mL) for 24 h. Total RNA was purified from the cells and subjected to real-time PCR analysis to determine the relative mRNA expression levels of *Slc5a5*, *Tpo*, *Duox2* and *Duoxa2*. mRNA levels were normalized against *Gapdh* levels and expressed as fold-change relative to the control. Data are presented as mean ± SD relative to the levels of PV (-) cells (n = 3). *p < 0.05 and ***p < 0.001, compared to PV (−) value.

In order to evaluate potential effects of PV on thyroid function, we examined the gene expression of the thyroid functional genes responsible for each step of thyroid hormone biosynthesis. When FRTL-5 cells were treated with PV, mRNA levels of *Slc5a5*, *Tpo*, *Duox2*, and *Duoxa2*, the functional genes involved in the transport and organification of iodide, were significantly suppressed in a dose-dependent manner (**Figure 6**). mRNA levels of *Gapdh* were not affected by PV (data not shown). These results suggest that PV may suppress active iodide uptake and oxidation.

## Discussion

As the precise pathogenesis of Hashimoto’s thyroiditis remains unclear, there is no established treatment that can effectively ameliorate the destructive thyroid autoimmune response or delay the progression of Hashimoto’s thyroiditis. The only available therapy for Hashimoto’s thyroiditis is to manage hormone levels with thyroid hormone replacement, such as levothyroxine, which, in most cases, needs to be taken for the rest of the patient’s life. Although the underlying mechanisms are unknown, PV has been empirically used to treat thyroid disorders, including Hashimoto’s thyroiditis, in traditional Chinese medicine for thousands of years. Only recently have modern clinical trials begun to gather evidence that supports the efficacy of PV to reduce the titers of TPO-Ab, TG-Ab, and Th 17 cells, a subset of pro-inflammatory T helper cells implicated in autoimmune and inflammatory disorders (2–8). In addition, *in vitro* studies have revealed an anti-inflammatory effect of PV by targeting NF-κB in stimulated macrophages (26, 27). In the current study, we further elucidated a novel anti-inflammatory activity of PV in thyrocytes that likely contributes to its therapeutic effect on Hashimoto’s thyroiditis.

Studies have suggested that the innate immune response in thyrocytes facilitates autosensitization that may eventually lead to thyroid autoimmunity (10, 12). PAMPs and DAMPs are the two main patterns of stimuli that cells encounter *in vivo* indicative of harmful microbial infection or cellular damage. The former refers to the molecules associated with pathogens such as lipopolysaccharides (LPS), peptidoglycans (PGN), and viral dsRNA that initiate and perpetuate infectious pathogen-induced inflammatory responses (28). The latter, in contrast, are host biomolecules derived from dying cells such as self dsDNA, heat-shock proteins, purine metabolites, and hyaluronan fragments that initiate and perpetuate non-infectious inflammatory responses. The innate immune responses triggered by PAMPs or DAMPs rapidly wall off the potentially dangerous events (i.e. infection or non-physiological cell death) before they are out of control. At the same time, the adaptive immune system is ready to be alerted if immunogenic antigens (either foreign or self) are present. However, inflammatory responses are double-edged swords, as they are indiscriminate and can damage healthy tissues. In the thyroids of predisposed individuals, inflammatory responses triggered by PAMPs or DAMPs might facilitate adaptive immune responses to autoantigens by attracting lymphocytes into the periphery, activating antigen presentation processes, and interfering with hormone synthesis (12). The inflammatory mediators can orchestrate together with the infiltrating lymphocytes to induce *de novo* formation of lymph follicles, and consequently convert the usually self-tolerant peripheral environment into an organ prone to autoimmunity. One reason that the thyroid is particularly prone to autoimmunity may be stemmed from thyrocytes’ vulnerability towards stimulations with PAMPs or DAMPs (12).

As we demonstrated in the current study, the gene expression of inflammatory mediators such as IFN-β, TNF-α and IL-6 surged in FRTL-5 cells stimulated by dsDNA or dsRNA, in parallel with activation of both NF-κB and IRF3 signaling pathways and nuclear changes indicative of apoptosis. In addition, molecules associated with antigen presentation pathways, such as MHC class I and LMP-2 were also significantly induced in thyrocytes after stimulation with dsDNA or dsRNA. This inflammatory phenotype, characterized by the activation of both NF-κB and IRF3 signaling pathways, induced production of pro-inflammatory cytokines, chemokines and type I interferons, cell-surface expression of molecule involved in antigen presentation process, has been repeatedly noticed after stimulation with both artificial nucleid acids and cytosol self-genomic DNA released from injured cells (10, 12, 14). These observations together emphasize that in response to PAMPs or DAMPs, thyrocytes would launch intense inflammatory and pro-immunogenic reactions.

Strikingly, PV annulled such inflammatory responses induced by dsDNA or dsRNA in thyrocytes, and provided an exceptional protective effect in the immune-activated thyrocytes. Such anti-inflammatory action of PV in thyrocytes, in addition to its previously documented immunomodulatory effects in macrophages (26, 27, 29), may help to explain the observed therapeutic efficacy of PV for Hashimoto’s thyroiditis. In turn, our new findings highlight that innate immune responses in thyrocytes triggered by dsDNA or dsRNA is likely a critical factor for precipitating thyroid autoimmunity, and herbal/compound agents (i.e. PV) that interfere with such innate immune responses in thyrocytes may serve as novel therapies for Hashimoto’s thyroiditis. So far the structures of the main chemical compounds in PV, identified on the basis of spectral analysis, include polygalacerebroside, ursolic acid, β-amyrin, quercetin, quercetin-3-O-β-D-galactoside, α-spinasterol, stigmasterol, β-sitosterol, and daucosterol (30). We did have tested a few of these purified chemicals, which showed similar effects as did the PV crude extract in the inflamed thyrocytes, however to a much-weakened extent. Thus, the overall effects of PV unlikely come from a single component but rather a natural combination. It will take a large-scale study to exhaust the combinations of the known chemicals in PV to elucidate its “core recipe”.

In addition to thyrocytes, innate immune response has been noticed in a variety of cell types upon stimulation with dsDNA or dsRNA (31–33). And we recently reported that self dsDNA-inducible inflammation in primary human keratinocytes played a role in the pathogenesis of psoriasis which is also an autoimmune disease (34). And given that PV has been used to treat all kinds of immune disorders and inflammation from viral infections, allergies, Crohn’s disease, diabetes, ulcerative colitis, gastroenteritis, to atherosclerosis, headache and cancers, the immune-modulating effects of PV may be true in many cells types (35–39). We next will confirm the effects of PV on normal human primary thyrocytes.

In addition, we found that PV significantly suppressed the mRNA levels of genes essential for iodide transport and organification, such as *Slc5a5, Tpo, Duox2 and Duoxa2*, in a dose-dependent manner in thyrocytes. Slc5a5 (or NIS) is known as the major plasma symporter responsible for iodine influx into thyrocytes (40). Oxidation of iodide further requires TPO and H_2_O_2_ generated by DUOX2 and DUOXA2 (41). Excessive iodide uptake and oxidation is generally believed to be a risk factor for thyroid autoimmunity due to harmful oxidative stress, and should be avoided in Hashimoto’s thyroiditis (42). Thus, PV may also function as an antioxidant in the thyroid by checking on excessive iodide uptake and oxidation. Further functional studies are needed to clarify how PV impacts iodide uptake and hormone production in thyrocytes. Before that, physicians should carefully monitor the thyroid hormone and TSH levels of patients receiving PV.

## Data Availability Statement

The raw data supporting the conclusions of this article will be made available by the authors, without undue reservation.

## Author Contributions

All authors contributed to the article and approved the submitted version. KS and YL designed and drafted the manuscript. The experimental procedures and data analysis were performed by FC, AK, YL, and MK. KS and AK gave experimental guidance.

## Funding

This work was supported by The Japan China Sasakawa Medical Fellowship, Guangdong medical science and Technology Research Fund Project (A2016439) and by JSPS KAKENHI Grant Numbers 15K09444, 17K08990 and 19K07875.

## Conflict of Interest

The authors declare that the research was conducted in the absence of any commercial or financial relationships that could be construed as a potential conflict of interest.
