# Supplementary material for: Innate Immune-Modulatory Activity of Prunella vulgaris in Thyrocytes Functions as a Potential Mechanism for Treating Hashimoto’s Thyroiditis
Source: Front Endocrinol (Lausanne). 2020 Nov 16;11:579648. doi: 10.3389/fendo.2020.579648 (PMC7701117; doi:10.3389/fendo.2020.579648)
Supplement: Supplementary file 1 [file DataSheet_1.docx]

**Supplementary Method**

**Trypan blue exclusion test of cell viability**

FRTL-5 cells were cultured in 24-well plates and treated with increasing concentrations of PV for 24 h, and then were trypsinized to 1 ml cell suspension for each well. Mix equal parts of 50 μl cell suspension and 50 μl 0.4% trypan blue dye by pipetting up and down. The mixture was analyzed immediately at room temperature. Total cell number and number of dead cells stained by trypan blue were counted on cell counting plates under a phase-contrast microscope.

**Supplementary Fig. 1.** **PV at working concentrations does not affect cell viability.** FRTL-5 cells were treated with increasing concentrations of PV arranged from 31.25 μg/mL to 500 μg/mL for 24 h. Cytotoxic effect of PV was then determined through trypan blue exclusion test of cell viability. The percentages of viable cells were normalized by that in the PV-untreated control cells. Data are presented as mean ± SD relative to the levels of control cells (n = 4). No significant difference in cell viability was found between PV-treated and PV-untreated cells. As a positive control, 1 μM actinomycin-D was added to the culture. ***: p< 0.001, compared to the control value.
