# Supplementary figures and images for: Innate Immune-Modulatory Activity of Prunella vulgaris in Thyrocytes Functions as a Potential Mechanism for Treating Hashimoto’s Thyroiditis
Source: Front Endocrinol (Lausanne). 2020 Nov 16;11:579648. doi: 10.3389/fendo.2020.579648 (PMC7701117; doi:10.3389/fendo.2020.579648)

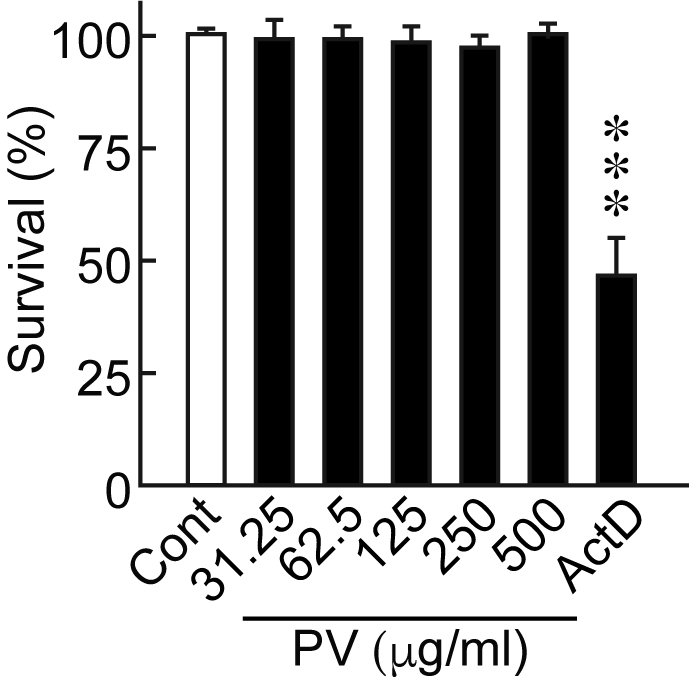

Supplement: Supplementary file 2 [file Image_1.jpeg]
